# Society Meetings

**Published:** 1896-11

**Authors:** 


### Society Meetings.

The Southern Surgical and Gynecological Association will hold its ninth annual meeting at Nashville, Tenn., Tuesday, Wednesday and Thursday, November 10, 11 and 12, 1896. The Nicholson house has been selected as headquarters for the association, and arrangements have been made with the Southern Passenger Association for reduced rates on the certificate plan. Those who contemplate attending the Pan-American Medical Congress, to be held in Mexico, November 16–19, will have time to do so after the meeting of the Southern Surgical and Gynecological Association. The rate of one fare for the round trip has been made on account of the Congress, stop-over privileges being allowed to holders of tickets to Mexico.

An elaborate program has been prepared under the direction of the secretary, Dr. W. E. B. Davis, of Montgomery, Ala.

---

The Lake Erie Medical Society held its regular quarterly meeting at the Crawford house, Cattaraugus, N. Y., Thursday, October 8, 1896. The officers were as follows: President, Dr. A. D. Lake, Gowanda; vice-president, Dr. N. A. Beardsley, Dunkirk; secretary and treasurer, Dr. W. W. Jones, Dayton; program committee: Dr. William N. Warren, North Collins; Dr. T. T. Clohessey, North Evans. The program was as follows: Address of welcome by Dr. Lattin, response by the president. Papers: (1) The differential diagnosis of spinal disease, Dr. Floyd S. Crego, Buffalo; (2) How shall we treat erysipelas? Dr. N. A. Beardsley, Dunkirk; (3) Pleurisy with effusion, Dr. George Lattin, Cattauraugus; (4) Presentation of cases.

---

The Medical Association of Central New York held its twenty-ninth annual meeting at Rochester, Tuesday, October 20, 1896, when the following officers were elected for the ensuing year: President, Edward B. Angell, Rochester; vice-president, William C. Krauss, Buffalo; second vice-president, F. H. Stephenson, Syracuse; secretary, C. A. Van Der Beek; treasurer, S. L. Elsner, Rochester.

The meeting in all respects was one of the best in the history of the association. The proceedings and papers, as usual, will appear in the Journal. The next meeting will be held at Buffalo, Tuesday, October 19, 1897. In order to join the association, attendance must be had upon two successive meetings and the payment of the initiation fee of $2. The yearly dues are $1, and in return all members receive the transactions.

